# Single-Station Coda Wave Interferometry: A Feasibility Study Using Machine Learning

**DOI:** 10.3390/ma14133451

**Published:** 2021-06-22

**Authors:** Erik H. Saenger, Claudia Finger, Sadegh Karimpouli, Pejman Tahmasebi

**Affiliations:** 1 Fachbereich Bau- und Umweltingenieurwesen, Bochum University of Applied Sciences, 44801 Bochum, Germany; 2 Fraunhofer IEG, Fraunhofer Research Institution for Energy Infrastructure and Geothermal Systems, 44801 Bochum, Germany; claudia.finger@ieg.fraunhofer.de; 3 Fakultät für Geowissenschaften, Ruhr-University Bochum, 44801 Bochum, Germany; 4 Mining Engineering Group, Faculty of Engineering, University of Zanjan, Zanjan 45371-38791, Iran; s.karimpouli@znu.ac.ir; 5 College of Engineering and Applied Science, University of Wyoming, Laramie, WY 82071, USA; ptahmase@uwyo.edu

**Keywords:** coda waves, reflection, machine learning, wave propagation, feasibility study

## Abstract

Coda wave interferometry usually is applied with pairs of stations analyzing the signal transmitted from one station to another. A feasibility study was performed to evaluate if one single station could be used. In this case, the reflected coda wave signal from a zone to be identified was analyzed. Finite-difference simulations of wave propagation were used to study whether ultrasonic measurements could be used to detect velocity changes in such a zone up to a depth of 1.6 m in a highly scattering medium. For this aim, 1D convolutional neural networks were used for prediction. The crack density, the crack length, and the intrinsic attenuation were varied in the considered background material. The influence of noise and the sensor width was elaborated as well. It was shown that, in general, the suggested single-station approach is a possible way to identify damage zones, and the method was robust against the studied variations. The suggested workflow also took advantage of machine-learning techniques, and can be transferred to the detection of defects in concrete structures.

## 1. Introduction

In Earth sciences, it is known that coda waves are sensitive to changes in the subsurface because the strong scattering that generates these waves causes them to repeatedly sample a limited region of space [1,2]. In contrast to the first arrival of an event, the influence on the waveforms due to changing subsurface features can be relatively high. However, the direct interpretation is challenging. Coda wave interferometry (CWI) is a technique that exploits tiny waveform changes in the coda to detect those variations of seismic properties in evolving media ([3] and references therein).

Moreover, the aging of concrete structures within the infrastructure (bridges, buildings, etc.) is a serious problem for many applications. Efficient monitoring should be performed to guarantee safety [4]. Ultrasonic transmission measurements have been used for decades to monitor concrete elements, mostly on a laboratory scale. Recently, CWI was introduced to civil engineering experiments [5]. A standard application setup for load tests of a concrete beam was described in a recent paper [6]: The material perturbations were detected around and in between a pair of a source and a receiver. They pointed out that the most common way to analyze coda waves is the correlation coefficient.

In this paper, the use of CWI for a source and the receiver at one single position is investigated. With the single-station approach, one mainly analyzes the reflected signal of a disturbed area in contrast to the approach described above ([6] and references therein), in which the authors concentrated on the transmitted signal. For this purpose, a numerical 2D wave-propagation setup is used, which allows us to create a relatively big number of recorded signal-traces that are suitable for machine learning (ML) techniques. The correlation coefficient is not calculated during this procedure. 

Thin rectilinear cracks (in which the crack length is on the order of the dominant wavelength) are distributed with a specific crack density in a homogeneous background media. This setup can mimic the wave-propagation regime in concrete, where the ratio of scatterers (e.g., grains) to the wavelength is similar. The main question to be answered in this study is: can a reduction of the background velocity (e.g., due to microcracks that are much smaller than the dominant wavelength) at a certain depth of the model be detected with CWI? For a real-world scenario, such microcracks are indicative of an evolving failure of the considered material.

To answer this question, a ML approach is used. Namely, convolutional neural networks (CNN) are applied to estimate the depth and reduced velocity background parameters. ML algorithms recently attracted much attention in different fields of sciences. In geosciences and geomaterials, they were used for either parameter estimation or classification (i.e., segmentation) [7,8,9,10]. In most of these studies, images of rocks or geomaterials were used as the input data for a network. However, in this study, the coda wave signals are used as 1D input data. This is more similar to speech or earthquake-detection problems, in which a signal is used for detection. In fact, CNNs can extract high-level features from the waveform of the coda waves by small-kernel convolutions, and then relate them in the output parameters. In this study, the aim is to use such an ability of the CNNs to extract concert characteristics from the waveform of a single-station reflected coda signal. The paper is structured as follows: First, the applied wave propagation technique and the chosen numerical setup are explained in detail. Second, the used ML approach and the networks are described. In a parameter study, the reliability of the method is checked to detect (a) the depth of the velocity change and (b) the amount of velocity reduction concerning crack density, crack length, noise level, and more. Finally, the findings and conclusions of the paper are discussed.

## 2. Numerical Setup and Machine-Learning Approach

### 2.1. Numerical Setup

A rotated staggered-grid (RSG) finite-difference (FD) scheme [11] was used to propagate the seismic wavefield in the forward simulations. The RSG used rotated finite-difference operators, leading to a distribution of modeling parameters in an elementary cell in which all components of one physical property were located only at one single position. This can be advantageous for modeling wave propagation in anisotropic media or complex media, including high-contrast discontinuities, as no averaging of elastic moduli is needed [12]. Due to the high density of scattering constituents, ultrasonic wave propagation in fractured media consists of a complex mixture of multiple scattering, mode conversion, and diffusive energy transport. With previous studies in 2D and 3D [13,14], it was demonstrated that the RSG-technique was well suited for such applications. The numerical accuracy for modeling wave propagation in a media with a single thin fracture was evaluated as well [15].

**Figure 1:**
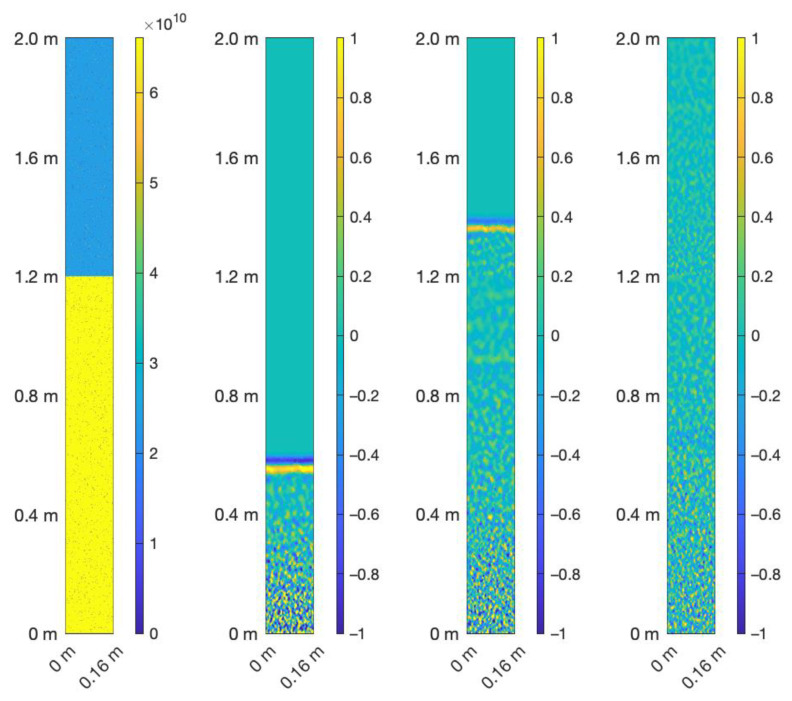
(**Left**) Elastic model of the fractured media as described in Section 2.1. The P-wave modulus c_44_ in Pascal is displayed. (**Right**) Three consecutive snapshots at t = 0.13 ms, t = 0.32 ms, and t = 0.77 ms of a propagating plane P-wave in the medium displayed on the left. The amplitude was normalized to the interval (−1, 1), but equal and comparable in all three subplots. Due to the relatively large amount of scattering, the coherent part of the reflected wave at the interface at d = 1.2 m became hardly detectable.

In Figure 1, a typical elastic model for the coda wave experiments of this study is shown on the left. The models, with a physical size of 0.16 m (x-direction) × 2.00 m (y-direction), consisted of 400 × 5000 grid points (GPs) with a spacing of d = 0.0004 m. In the first step, a homogeneous material was created with a P-wave velocity of V_P_ = 5100 m/s, an S-wave velocity of V_S_ = 2944 m/s, and a density of 2540 kg/m^3^. In a second step, thin (1 GP) rectilinear cracks (elastic moduli were set to zero) with a length of L = 2l (in Figure 1: L = 10 GP = 0.004 m) and a crack density *ρ* (in Figure 1 *ρ* is 0.1) were inserted into this media. The crack density was defined as in [16]:

(1) $$\rho =\frac{1}{A}\overset{n}{\underset{k=1}{\sum }}l_{k}^{2}$$

where *n* is the number of cracks and *A* is the representative area. As the third step, the velocities V_P_ and V_S_ were reduced from a randomly chosen depth d in the x-direction (used interval: 0 m < d < 1.6 m) by an arbitrary chosen factor r (used interval: 1 > r > 0.5). In the example displayed in Figure 1, d = 1.2 m and r = 0.6 was used. It is important to note that the modeling experiments were performed with periodic boundary conditions in the horizontal direction. For this reason, the elastic models were generated with this periodicity as well. In addition, a free surface was implemented at the bottom, and absorbing boundary conditions were applied at the top of the modeling domain.

**Figure 2:**
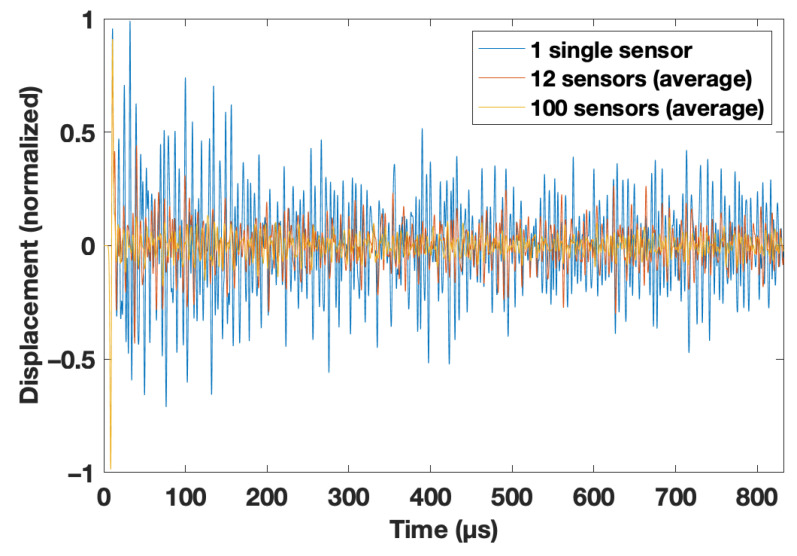
Recorded traces at the bottom of the model at y = 0 m. Three cases are displayed: Signal for a single receiver (blue); averaged signal for 12 neighboring central sensors with a total width of 1.92 cm and averaged signal of 100 sensors equally distributed over the full width of the model (16 cm).

For all simulations, a wavelet (second derivative of a Gaussian) with a central frequency of f_c_ = 100 kHz was used. A body-force line source was placed at the bottom of the model (y = 0 m). The plane P-wave generated in this way propagated through the fractured medium (see Figure 1, right). The time increment was set to 6.4 × 10^−8^ s to ensure stability, and 100 equidistant receivers, with a spacing of 4 × 0.0004 m, recorded the displacement during the simulation with 12,500 timesteps (see Figure 2) at the bottom of the model (y = 0 m). Therefore, the source and receivers were in the same position to record reflected coda waves. On a midsize Linux cluster, the simulations took roughly 12 min each. An overview of all simulations is given in Table 1.

For one series of simulations, intrinsic attenuation was implemented in the applied FD scheme [17]. With the parameters $Y_{1}^{11}=3.8979\;\mathrm{GPa}$, $Y_{1}^{44}=1.2989\;\mathrm{GPa}$, and $\omega_{1}=628\;\mathrm{kHz}$, a frequency-dependent attenuation with a single Maxwell body with a minimal Q = 33 at f_fund_ = 100 kHz was simulated (equal to the central frequency of the wavelet).

**Figure 3:**
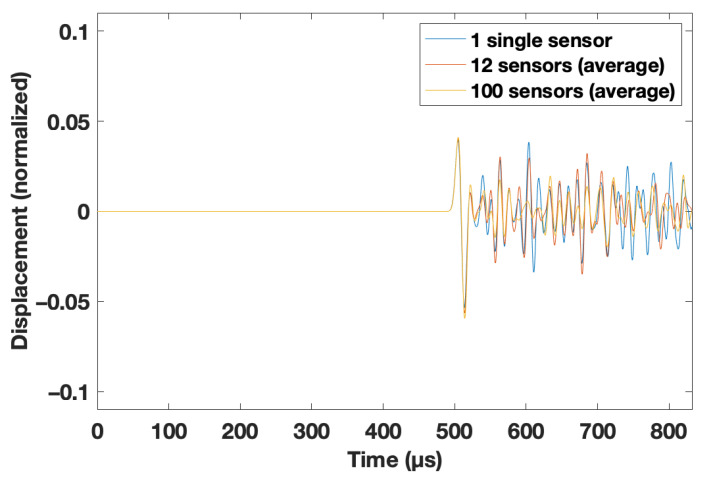
Processed traces at the bottom of the model at y = 0 m. The traces of the simulation with d = 1.2 m and r = 0.6 were subtracted by the recorded traces for the simulation with r = 1.0 (see text for details).

When the wavelength of the propagating wave was in the order of the heterogeneities (i.e., the thin cracks), both Rayleigh and Mie scattering occurred [18], and was very strong in the considered cases of this study. A standard imaging technique thus could not be applied here because the reflected wave was not easily detectable in a single, or in the averaged traces as shown in Figure 2. This only becomes feasible when the traces of the medium with no velocity reduction are known. Furthermore, this event was visible when the recordings at the position y = 0 m for the starting model (r = 1.0) were subtracted from the signals recorded with 0 m < d < 1.6 m and 1 < r < 0.5. This is shown for d = 1.2 m and r = 0.6 in Figure 3.

**Figure 4:**
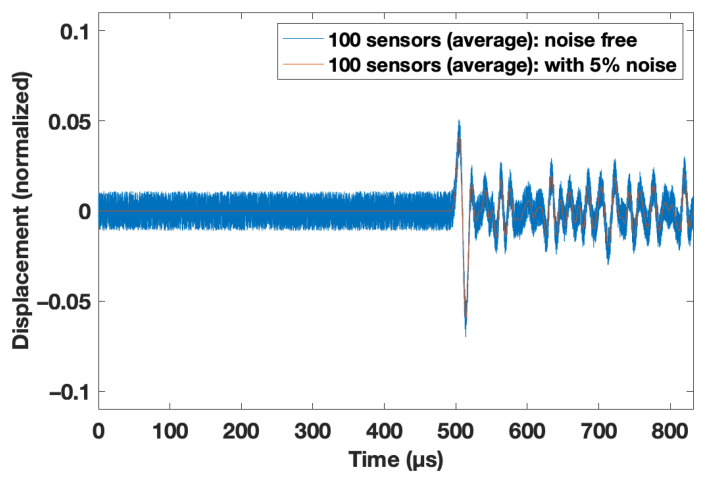
Processed traces at the bottom of the model at y = 0 m. The traces of the simulation with d = 1.2 m and r = 0.6 were subtracted by the recorded traces for the simulation with r = 1.0. To come closer to a realistic scenario, different noise levels were added (for details, see Section 2.1).

In practical applications, there is always a certain amount of noise. As such, several noise levels (5%, 10%, and 20%) were considered. For this purpose, random values were added to the trace in the range of ±5%, 10%, or 20% of the maximum coda signal amplitude. An example is given in Figure 4.

### 2.2. Machine Learning

**Figure 5:**
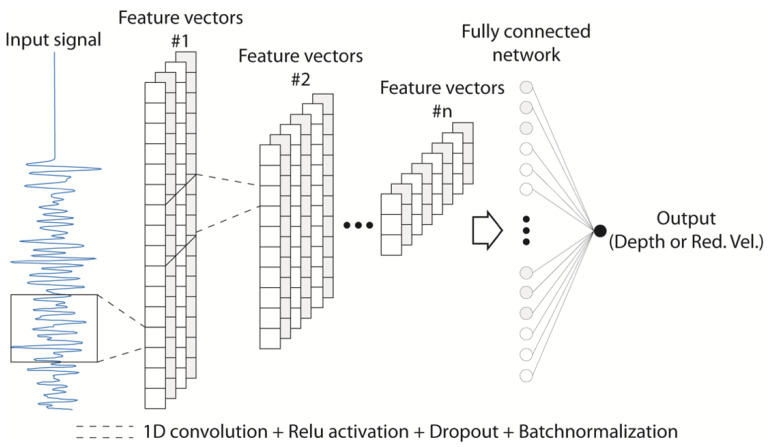
The general architecture used in this study. For more information about the network, please refer to Table 2.

In this study, two individual CNNs were used each for depth and reduced velocity predictions (Figure 5). The input of the network was a signal recorded at the bottom (y = 0 m) of the model with a length of 2500 cells (with a time step of 3.2 × 10^−7^ s). Four feature vectors with #n channels were extracted from the input signal, as shown in Figure 5. To extract each feature vector, the vector in the last layer was convolved by a 1D convolutional layer. Consequently, convolved vectors were downsampled by 1D max-pooling, which was followed by dropout and batch-normalization layers (Figure 5) [7]. This architecture extracted high-level features of the input coda signal and produced a small-size feature vector, which was highly informative compared to the input signal. This vector was connected to the output using a fully connected layer followed by dropout and batch-normalization layers. More details are shown in Table 2.

In each model setup (Table 1), more than 1024 signals with varied d and r were simulated. A soft-clipping normalization was applied to normalize the amplitude of the signals into (0, 1) (see [19]):

(2) $$S_{n}=\frac{1}{1+e^{-kS_{o}}}$$

where $S_{o}$ and $S_{n}$ are original and normalized signals and k is chosen empirically, based on the maximum amplitude in the signal (here it is 16). The depth and reduced velocity values were also normalized in the range of (0, 1). All data were divided into 75% and 25% as training and validation data, respectively. Validation data also were used as test data, since they were not learned by the network during the training phase [7]. Mean square error (MSE) and coefficient of determination (R^2^) were used as two widely accepted criteria for the evaluation of estimations as follows:

(3) $$MSE=\frac{1}{n}\overset{n}{\underset{i=1}{\sum }}{\left(Y_{i}-{\hat{Y}}_{i}\right)}^{2}$$

(4) $$R^{2}=1-\frac{SS_{res}}{SS_{tot}}$$

where Y and $\hat{Y}$ are original and estimated parameters for n data. In addition, $SS_{tot}$ and $SS_{res}$ are the total and residual sum of squares errors regarding mean and predicted parameters, respectively.

## 3. Results

**Figure 6:**
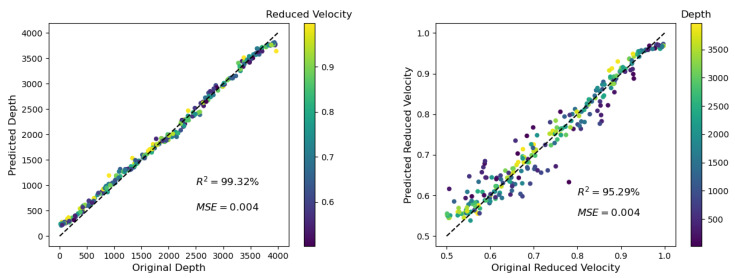
(**Left**) Depth prediction of the ML algorithm for the example described in Section 3.1. The reduced velocity r is color-coded and has no unit (see Section 2.1). (**Right**) Corresponding velocity prediction: R^2^ is the coefficient of determination, and MSE is the mean square error (for details, see Section 2.2). The depth d is color-coded and is given in meters (m).

In this section, the results of the numerical study are described in detail. For each case, the predicted depth and reduced velocity were compared with the actual values. For a better interpretation, the second quantity (i.e., reduced velocity or depth) included is always color-coded in the corresponding plots (see, e.g., Figure 6).

### 3.1. Case1: Basic Example (Average 100 Traces, No Noise, ρ = 0.1, L = 10)

For this basic example, 100 traces recorded at the bottom of the model (see Figure 1 and Figure 3) were averaged. The background model had a crack density *ρ* of 0.1 with a crack length of 0.004 m (i.e., 10 grid points). No noise was added. With the setup described in detail in Section 2, the ratio of fundamental wavelength to crack length was roughly 12.

It can be observed that the predicted depth d of the velocity change was for the full range of possible depths very high (Figure 6, left; R^2^ = 99.32% and MSE = 0.004). The predicted velocity reduction r was still very good, but not as good as for the predicted depth d (Figure 6, right, R^2^ = 95.29% and MSE = 0.004). Interestingly, a detailed inspection of the predicted velocity change indicated that this prediction became more inaccurate for situations in which the velocity change was closer to the source position (see Figure 6, right; the darker-colored dots are farther away from the optimal dashed line than the lighter-colored dots). This case was due to a shallow reflector creating a longer nonzero signal with much more information than a signal for a deep reflector. Learning such a complex signal (shallow d) is more difficult than learning a simple signal (deep d) for ML.

### 3.2. Case 2: Variation of the Crack Density

**Figure 7:**
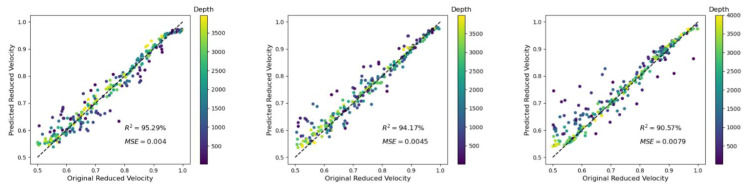
(**Left**) Velocity prediction for a crack density *ρ* of 0.1 (same as Figure 6, right); (**center**) for a crack density *ρ* of 0.2; and (**right**) *ρ* = 0.3. The accuracy slightly decreased with increasing crack density. Moreover, it was observed that the largest errors occurred for relatively low depths.

In this example, the influence of the crack density on our depth and velocity predictions was considered. In Figure 7, there is a focus on the prediction of the velocity reduction, as the depth prediction was not so disturbed by the variation of the crack density *ρ* (*R*^2^ = 99.32%, 99.31%, and 99.13% for *ρ* = 0.1, 0.2, and 0.3, respectively). However, as shown in Figure 7, the inaccurate prediction of the reduced velocity for the case of relatively shallow depths (darker dots in Figure 7) became stronger when the crack density was increasing.

### 3.3. Case 3: Variation of Crack Length 

**Figure 8:**
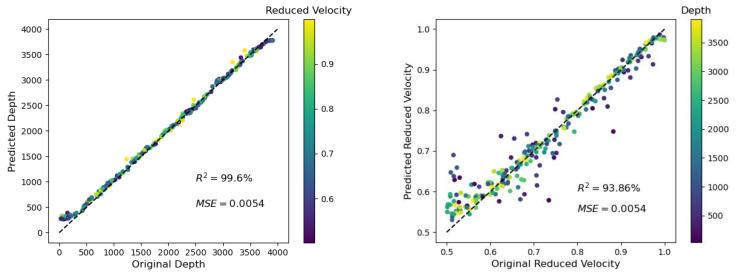
Depth prediction as shown in Figure 6, but for a crack length of L = 40 grid points instead of 10 grid points.

In Figure 8, an example in which the crack length was increased from L = 10 to L = 40 grid points is shown. Here, the ratio of wavelength to crack length was decreasing, and a decreasing accuracy for relatively shallow depths d for the velocity reduction r was observed.

### 3.4. Case 4: Influence of Noise

**Figure 9:**
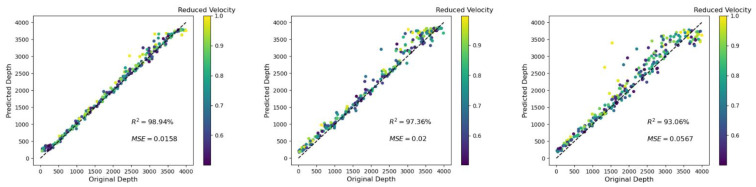
Depth predictions for the case of a crack density of *ρ* = 0.3 and L = 10 GPs with an increasing noise level of 5%, 10%, and 20% (**left** to **right**). The reduced velocity r is color-coded and will had no unit (see Section 2.1).

The influence of different noise levels was studied for several scenarios (Table 3). Due to similar trends of the results, two examples are shown here: (a) for a crack density of *ρ* = 0.3 and a crack length L = 10 grid points; and (b) for a crack density of *ρ* = 0.1 and a crack length L = 40 grid points. As described above, both examples gave relatively good predictions for the case without noise. Considering the depth prediction for the first case (*ρ* = 0.3 and L = 10 GPs), adding noise decreased the accuracy for relatively deep depth, but the results were in general still acceptable (Figure 9).

**Figure 10:**
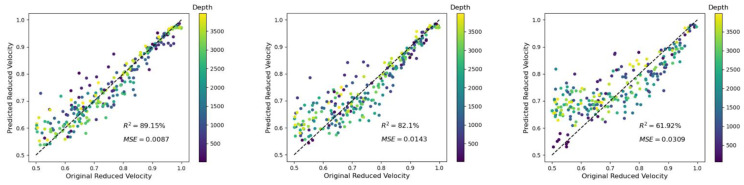
Velocity-reduction predictions for the case of a crack density of *ρ* = 0.1 and L = 40 GPs with an increasing noise level of 5%, 10%, and 20% (**left** to **right**). The depth d is color-coded and is shown in meters (m).

The predictions of the ML algorithm for the reduced velocity in the case of relatively high noise and a relatively high crack length (L = 40 GPs) brought the method close to its limits (see Figure 10). For the case of a noise level of 20%, the predicted reduced velocity was not as good as for the other examples given in this study (R^2^ = 61.92%).

### 3.5. Case 5: Application of Different Averaging Techniques at the Bottom of the Model

**Figure 11:**
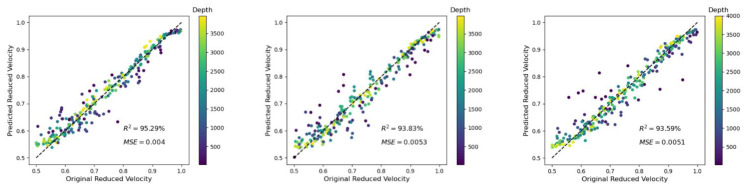
Influence of the predicted reduced velocity on averaging of neighboring sensors (distance is equal to 4 times the grid spacing) for a crack density *ρ* = 0.1 and L = 10 GPs. (**Left** to **right**) Averaging the signal of 100 sensors, of 12 sensors, and the analysis of one single sensor. The depth d is color-coded and is given in meters (m).

Finite difference simulations have a given grid spacing, and at every grid point, a sensor (or receiver) can be placed. In the case of averaging signals of neighboring sensors, the influence of the sensor width (i.e., sensor size) was considered. For a realistic comparison of ultrasonic experiments with wave-propagation simulations, this should be kept in mind because very often the sensor size cannot be neglected. In the used setup, trace recording from a single receiver was equivalent to a sensor width of 0.0004 m (i.e., the grid spacing). Averaging of 12 neighboring sensors and 100 neighboring sensors corresponded to sensor widths of 0.192 m and 0.16 m, respectively. As expected, there was only a minor influence on the prediction of the reduced velocity (see Figure 11).

### 3.6. Case 6: The Effect of Intrinsic Attenuation

**Figure 12:**
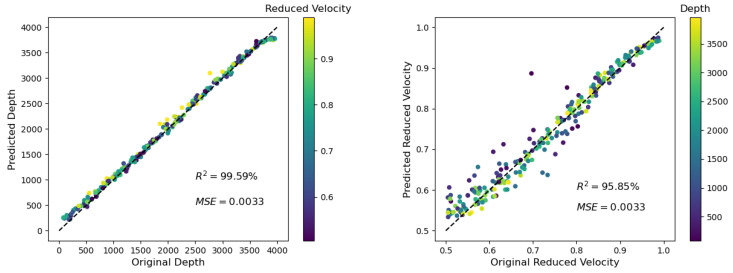
Depth prediction as shown in Figure 6 (*ρ* = 0.1, L = 10 GPs), but for simulations with a minimum quality factor Q of 33, which mimicked the attenuation in concrete (for details, see Section 2.1 and Section 3.6).

As reported in [20], ultrasound is attenuated in cement-based materials, and can be compared with the attenuation observed in geomaterials; e.g., granite. Therefore, the influence of intrinsic attenuation was also studied for the application of the presented method. It revealed that a relatively strong attenuation (minimum quality factor of Q = 33 for the used frequencies) did not have a significant influence on the predictions. The coefficient of determination R^2^ was changed from 99.32% to 99.59% and from 95.29 to 95.85% (see Figure 6 and Figure 12) for the predicted depth and the reduced velocity, respectively.

## 4. Discussion

In Section 3, the influence of crack density *ρ*, crack length L, noise level, sensor width, and attenuation on the predicted depth d and reduced velocity r were considered. Table 3 presents a complete list of all results (a few are not displayed in a separate figure). In general, the proposed numerical parameter study using one single station to apply coda wave interferometry can be regarded as a successful confirmation of the applied method to characterize a velocity reduction in an unknown depth. This velocity reduction may indicate damage on the microscale (i.e., microcracking in which the cracks are much smaller than the wavelength). In contrast to the classical coda wave interferometry technique (i.e., [6]), the reflected signal of a material change is analyzed instead of the transmitted signal.

The accuracy of the predicted depth and the reduced velocity could be reduced by several factors, which included: increasing crack density, increasing crack length, and most prominently, increasing noise. The influence of intrinsic attenuation and averaged traces (i.e., sensor width) could be regarded as minor. Moreover, the accuracy for the predicted depth was always very high, whereas the reduced velocity itself was predicted with a lower accuracy (but still acceptable). Of course, strong noise had a significant influence on the quality of the predictions (see, e.g., Figure 10).

The chosen setup could be used to evaluate if a single-station CWI approach could be applied to find a damage zone in concrete. The background velocity of the cracked media and the fundamental frequency of the source wavelet were close to the values used in [6]. The crack length could be compared to several grain-size distributions and the size of air inclusions in real concrete. Therefore, in this study, it was shown that with a single station, it was possible to predict small-scale damages in a depth down to 2 m in concrete with a trained CNN.

This may be true for a more conventional investigation of the analyzed signal shown in Figure 4. An obvious thought would be to analyze the arrival time and the amplitude of the first change of the coda wave (i.e., the first nonzero amplitude of the subtracted signals from the disturbing case to the undisturbed case). For real experiments, this would be a relatively easy opportunity to estimate the depth (i.e., by picking the arrival time) and the reduced velocity (i.e., by analyzing the amplitude with the law for the reflection coefficient [21]). However, for this feasibility study, it was decided to use a CNN, as it was independent of a picking algorithm, which usually needs a significant amount of human interaction [22]. Moreover, the CNN used the full recorded trace, which contained more information that might be hidden by the complex scattering regime (the wavelength was in the same order as the scatterers; compare with Section 2).

Of course, more simulations (both in 2D and 3D) must be performed to train the machine-learning system on a specific concrete in a real experiment to be considered. This would be time-consuming, as especially 3D simulations are very demanding concerning computational resources. On the other hand, once the CNN is trained, the single-station coda wave analysis for damage detection can be performed in real-time, as the ML method works fast and without any human interaction.

## 5. Conclusions

In this paper, a parameter study was presented using ML to check whether a reflected coda wave technique could be used to detect and characterize a damaged zone with a single transducer (i.e., station). For this purpose, a numerical setup was built to systematically study the influence of multiple scatterers, noise, intrinsic attenuation, and sensor width.

In general, all these factors influenced the accuracy of the approach, but the accuracy of the predicted depth of the damaged zone (i.e., the depth of the zone with a reduced velocity) was especially high. In addition, the amount of the velocity reduction could be predicted with sufficient accuracy. It can be summarized that the most influencing factors were, as expected, higher levels of noise. For practical applications, one should make sure to record high-quality data with a relatively low noise level.

The investigation can be regarded as a successful feasibility study using one single station for coda wave interferometry. The method is ready to be applied to real experiments, and should be used complementary to traditional coda wave interferometry based on transmission. The fact that a reflector can be identified and characterized with the suggested coda wave technique suggests the application of more advanced imaging techniques in this context.

## Figures and Tables

**Table 1:** Overview of all simulations.

Fixed Properties	Random Properties	Number of Performed Simulations
*ρ* = 0.1, L = 10 GPs	Depth d = 5–4000 GPs; velocity reduction r = 0.5–1	>1024
*ρ* = 0.2; L = 10 GPs	Depth d = 5–4000 GPs; velocity reduction r = 0.5–1	>1024
*ρ* = 0.3; L = 10 GPs	Depth d = 5–4000 GPs; velocity reduction r = 0.5–1	>1024
*ρ* = 0.1; L = 40 GPs	Depth d = 5–4000 GPs; velocity reduction r = 0.5–1	>1024
*ρ* = 0.1, L = 10 GPs, Q_min_ = 33	Depth d = 5–4000 GPs; velocity reduction r = 0.5–1	>1024

**Table 2:** The architecture of the CNNs used in this study (see Figure 5) (ch: number of channels; k: kernel size; ReLU, Tanh, and Sigmoid: activation functions).

	Output	Depth	Reduced Velocity	Inner Networks
Layer				
Conv-1D		16 ch, 3 k, ReLU	128 ch, 3 k, ReLU	Feature vector #1
Maxpooling-1D		Pool size: 2		
Dropout		10%		
Batch-normalization				
Conv-1D		32 ch, 3 k, ReLU	128 ch, 3 k, ReLU	Feature vector #2
Maxpooling-1D		Pool size: 2		
Dropout		10%		
Batch-normalization				
Conv-1D		64 ch, 3 k, ReLU	256 ch, 3 k, ReLU	Feature vector #3
Maxpooling-1D		Pool size: 2		
Dropout		10%		
Batch-normalization				
Conv-1D		128 ch, 3 k, ReLU	256 ch, 3 k, ReLU	Feature vector #4
Maxpooling-1D		Pool size: 2		
Dropout		10%		
Batch-normalization				
Dense		1024 ch, Tanh	1024 ch, Tanh	Fully connected network
Dropout		30%		
Batch-normalization				
Dense		1 ch, Sigmoid	1 ch, Sigmoid	Output

**Table 3:** Overview of all ML results.

Model Setup				Predicted Depth		Predicted Reduced Velocity	
Crack Density *ρ*	Crack Length L	Traces	Specifics	R^2^%	MSE	R^2^%	MSE
				Training		Training	
				Validation/Test		Validation/Test	
0.1	10 GPs	100	-	99.56	0.0028	97.36	0.0032
				99.32	0.0040	95.29	0.0040
0.1	10 GPs	100	5% noise	99.48	0.0018	97.06	0.0026
				99.07	0.0033	96.04	0.0033
0.1	10 GPs	100	10% noise	99.25	0.0043	92.04	0.0056
				98.54	0.0079	90.12	0.0079
0.1	10 GPs	100	20% noise	99.15	0.0096	86.38	0.0085
				98.00	0.0132	84.57	0.0132
0.2	10 GPs	100	-	99.63	0.0035	96.34	0.0021
				99.31	0.0045	94.17	0.0045
0.2	10 GPs	100	5% noise	97.89	0.0111	85.73	0.0115
				97.23	0.0127	84.68	0.0127
0.2	10 GPs	100	10% noise	96.88	0.0114	76.12	0.0135
				96.40	0.0205	74.42	0.0205
0.2	10 GPs	100	20% noise	91.56	0.0223	59.35	0.0256
				90.81	0.0372	56.19	0.0372
0.3	10 GPs	100	-	99.48	0.0062	92.03	0.0053
				99.13	0.0079	90.57	0.0079
0.3	10 GPs	100	5% noise	99.36	0.0125	80.95	0.0112
				98.94	0.0158	80.65	0.0158
0.3	10 GPs	100	10% noise	97.78	0.0165	76.98	0.0189
				97.36	0.0200	74.37	0.0200
0.3	10 GPs	100	20% noise	93.65	0.0428	35.37	0.0550
				93.06	0.0567	32.30	0.0567
0.1	40 GPs	100	-	99.68	0.0048	94.50	0.0051
				99.60	0.0054	93.86	0.0054
0.1	40 GPs	100	5% noise	98.84	0.0068	90.28	0.0061
				98.06	0.0087	89.15	0.0087
0.1	40 GPs	100	10% noise	98.01	0.0095	84.45	0.0132
				96.58	0.0143	82.10	0.0143
0.1	40 GPs	100	20% noise	92.95	0.0268	65.08	0.0243
				92.39	0.0309	61.92	0.0309
0.1	10 GPs	12	-	99.53	0.0019	94.57	0.0037
				99.32	0.0053	93.83	0.0053
0.1	10 GPs	1	-	99.85	0.0032	93.86	0.0046
				99.56	0.0051	93.59	0.0051
0.1	10 GPs	100	Q_min_ = 33	99.68	0.0016	96.42	0.0030
				99.59	0.0033	95.85	0.0033

## Data Availability

The data are available upon request from the corresponding author.
